# Motor Cortex Neurostimulation Technologies for Chronic Post-stroke Pain: Implications of Tissue Damage on Stimulation Currents

**DOI:** 10.3389/fnhum.2016.00545

**Published:** 2016-11-09

**Authors:** Anthony T. O’Brien, Rivadavio Amorim, R. Jarrett Rushmore, Uri Eden, Linda Afifi, Laura Dipietro, Timothy Wagner, Antoni Valero-Cabré

**Affiliations:** ^1^Neuromodulation Lab and Center for Clinical Research and Learning – Department of Physical Medicine and Rehabilitation, Spaulding Rehabilitation Hospital, Harvard Medical School, BostonMA, USA; ^2^Laboratory of Cerebral Dynamics, Plasticity and Rehabilitation, Boston University School of Medicine, BostonMA, USA; ^3^Department of Anatomy and Neurobiology, Boston University School of Medicine, BostonMA, USA; ^4^Department of Mathematics and Statistics, Boston University, BostonMA, USA; ^5^Highland Instruments, CambridgeMA, USA; ^6^Division of Health Sciences and Technology, Harvard Medical School/Massachusetts Institute of Technology, BostonMA, USA; ^7^Université Pierre et Marie Curie, CNRS UMR 7225-INSERM U1127, Institut du Cerveau et la Moelle EpinièreParis, France; ^8^Cognitive Neuroscience and Information Technology Research Program, Open University of CataloniaBarcelona, Spain

**Keywords:** epidural brain stimulation, transcranial magnetic stimulation, transcranial direct current stimulation, motor cortex, neurological model, stroke, pain, analgesia

## Abstract

**Background:** Central post stroke pain (CPSP) is a highly refractory syndrome that can occur after stroke. Primary motor cortex (M1) brain stimulation using epidural brain stimulation (EBS), transcranial magnetic stimulation (TMS), and transcranial direct current stimulation (tDCS) have been explored as potential therapies for CPSP. These techniques have demonstrated variable clinical efficacy. It is hypothesized that changes in the stimulating currents that are caused by stroke-induced changes in brain tissue conductivity limit the efficacy of these techniques.

**Methods:** We generated MRI-guided finite element models of the current density distributions in the human head and brain with and without chronic focal cortical infarctions during EBS, TMS, and tDCS. We studied the change in the stimulating current density distributions’ magnitude, orientation, and maxima locations between the different models.

**Results:** Changes in electrical properties at stroke boundaries altered the distribution of stimulation currents in magnitude, location, and orientation. Current density magnitude alterations were larger for the non-invasive techniques (i.e., tDCS and TMS) than for EBS. Nonetheless, the lesion also altered currents during EBS. The spatial shift of peak current density, relative to the size of the stimulation source, was largest for EBS.

**Conclusion:** In order to maximize therapeutic efficiency, neurostimulation trials need to account for the impact of anatomically disrupted neural tissues on the location, orientation, and magnitude of exogenously applied currents. The relative current-neuronal structure should be considered when planning stimulation treatment, especially across techniques (e.g., using TMS to predict EBS response). We postulate that the effects of altered tissue properties in stroke regions may impact stimulation induced analgesic effects and/or lead to highly variable outcomes during brain stimulation treatments in CPSP.

## Introduction

Central post stroke pain (CPSP) results from stroke lesions to any region of the somatosensory pathway (Klit et al., 2009; Kumar et al., 2009; Creutzfeldt et al., 2012; Mozaffarian et al., 2015). Between 8 and 25% of the ~18 M/year new cases of stroke develop CPSP (Strong et al., 2007; Klit et al., 2015). CPSP leads to poor quality of life (Kumar and Soni, 2009; Oh and Seo, 2015). Patients are often refractory to pharmacotherapy and can become drug dependent (Kumar and Soni, 2009). Such limitations have motivated researchers to explore brain stimulation therapies to treat CPSP.

Epidural Brain Stimulation (EBS), Transcranial Magnetic Stimulation (TMS), and Transcranial Direct Current Stimulation (tDCS) have all been investigated. Stimulation of primary motor cortex (M1) appears to be the most effective cortical target (Nguyen et al., 1999; Kumar and Soni, 2009; Hirabayashi et al., 2011; DosSantos et al., 2012; Fregni et al., 2014; Brietzke et al., 2015; Cioato et al., 2015; Morishita et al., 2015; Oh and Seo, 2015). Analgesia is believed to be achieved through the stimulation of M1-thalmic relays to reduce hyperactivity in thalamic linked pain networks (Tsubokawa et al., 1993; Mertens et al., 1999; Khedr et al., 2005; Garcia-Larrea and Peyron, 2007; Peyron et al., 2007; Lima and Fregni, 2008; Nguyen et al., 2008; Fontaine et al., 2009; Lefaucheur et al., 2009; Ohn et al., 2012; Bae et al., 2014; Hasan et al., 2014; Lefaucheur, 2016).

While EBS, TMS, and tDCS have shown some clinical success in treating CPSP, high variability across studies has impeded their widespread acceptance (Mertens et al., 1999; Lefaucheur et al., 2004, 2009; Lima and Fregni, 2008; Nguyen et al., 2008; Fontaine et al., 2009; DosSantos et al., 2012; Bae et al., 2014; Lefaucheur, 2016). Upward of 30% of EBS patients do not respond to stimulation (Tsubokawa et al., 1993; Katayama et al., 1998; Mertens et al., 1999; Nguyen et al., 1999). However, it should be noted that this is highly dependent on patient characteristics, and even lower response rates have been reported in certain patient classes (Katayama et al., 1998). Meta-analyses by O’Connell et al. (2014) and Vaseghi et al. (2014) demonstrated limited evidence supporting the use of TMS or tDCS in chronic pain and CPSP. Vaseghi et al. (2014), who focused on tDCS, commented that stimulation could induce significant analgesic effects, but due to the heterogeneity across studies it is difficult to support its use in chronic pain (O’Connell et al., 2014; Vaseghi et al., 2014).

Such variable levels of efficacy have been associated with several factors such as lesion location and extent, the impact of altered neuronal excitability, and the shrinkage of gray and white matter (Hossman, 2009). Infarction based changes in brain tissue conductivity could also impact stimulation based CPSP treatments. Necrotic brain tissue in the infarction region is phagocytized by inflammatory cells and replaced by a cerebral spinal fluid (CSF) (De Girolami et al., 1999). CSF produces a sixfold increase in the tissues’ electrical conductivity and a drastic disruption of the tissue geometry (Yunokuchi et al., 1998; Jacobs et al., 2001; Brown et al., 2003; Soltanian-Zadeh et al., 2003; Wagner et al., 2004, 2006, 2007a; Harris-Love and Cohen, 2006). Such altered electrical tissue properties have been shown to perturb the stimulating currents during TMS and tDCS (Wagner et al., 2006, 2007b, 2009).

Nevertheless, as emphasized by Plow and others, the role of such variables in influencing the distribution of current fields and ultimately impacting therapeutic efficacy in focally injured brain models needs further consideration, and remains to be compared across different brain stimulation techniques (Plow et al., 2009). Comparisons across stimulation techniques, which differ by electrode/source size, focality, invasiveness, proximity to lesion borders and specific features of the delivered electrical currents, are fundamental to evaluating and optimizing their clinical use (Plow et al., 2009). Furthermore, this comparative information is important for assessing the use of non-invasive stimulation techniques to identify responders to CPSP stimulation treatments prior to implanting invasive stimulation devices (Khedr et al., 2005; Lefaucheur, 2013, 2016).

The aim of this study is to determine how infarctions and/or complex neuroanatomy could alter the neurostimulation currents of the three primary neurostimulation techniques used in CPSP and potentially impact their clinical significance.

## Materials and Methods

Simplified magnetic resonance imaging (MRI) guided Finite Element Models (FEMs) of the stimulating current density distributions elicited through EBS, TMS, and tDCS were generated. The models were generated following methods previously outlined (Wagner et al., 2004, 2007b), and following foundational physics reviewed in the appendix of Wagner et al. (2014).

Briefly, we developed a FEM head/brain model with a healthy brain (developed from the MRI of a 38-year-old male) and a second model that included a circumscribed frontal cortical lesion within the head, specifically modeling a middle cerebral artery (MCA) based occlusion (Wagner et al., 2004). For simplification purposes, we focused on the comparison across stimulation techniques most commonly used to treat CPSP, and thus the head models did not include sulci and gyri, but only the presence of the lesion. Furthermore, we assumed static fields during stimulation for tDCS and EBS and sinusoidal steady state solutions during TMS.

The models were developed with Ansoft’s Maxwell software (Ansoft Inc, Pittsburg, PA, USA). We specifically solved a modified magnetic diffusion equation for the TMS models:

$$\nabla \times \left(\frac{1}{\sigma (\omega )+j\omega \varepsilon (\omega )}\nabla \times \overset{\wedge }{\text{H}}\right)=-j\omega \mu \overset{\wedge }{\text{H}}$$

where H is the magnetic field in phasor form, sigma the tissue conductivity, epsilon the tissue permittivity, and omega the angular frequency of the source. The Ansoft package numerically solves the problem via a modified T-Ω method (Wagner et al., 2004). For the tDCS and EBS models, the Ansoft FEM solver was set to solve for the current densities in terms of the electric potential (ϕ), by solving the equation: ∇⋅(σ_*i*_∇ϕ) = 0, where σ_i_ is the conductivity of the tissue (Ansoft) (Wagner et al., 2007b). For each model, the Ansoft FEM solver was set to follow an adaptive iterative process with convergence limits determined by the energy error in the system, further detailed in Ansoft (2002, 2005). The criterion for model convergence was defined as an energy error below 1.0% (Wagner et al., 2004, 2007a).

The current source device parameters correspond to those typically used in clinical studies and trials (Brown et al., 2006; Fregni et al., 2007; Lima and Fregni, 2008). The TMS source current was set as in prior modeling studies at 5 kHz with a 1.8 × 10^3^ A peak current on a figure-of-eight coil with two 3.5 cm radius copper windings (Wagner et al., 2004). The tDCS source current was set at 1 mA across a 5 × 7 cm anode (on a scalp area overlying the motor strip) and cathode (above the contralateral *orbital*) (Wagner et al., 2007a). The EBS source was set at 1 mA, with the anode and cathode placed above the M1 (18 mm inter-contact distance, 1 mm radius) (Brown et al., 2006). Note that those EBS parameters are based on *Adtech* 1 mm radius electrodes mounted on a 3 × 3 grid over an 18 × 18 mm area (where the inner row is inactive) which generates three separate bipolar arrangements (distanced 18 mm)- (Adtech Medical Instrument Corp) (Brown et al., 2003).

While, we used a 1 mA source magnitude for EBS, it should be noted that the EBS solutions are linear in the region of interest and simple multiplicative scaling can be used to account for varied source magnitudes (Woodson and Melcher, 1968; Zahn, 2003; Wagner et al., 2014). Furthermore, as the EBS electrostatic solutions are addressable by superposition, we focused on one bipolar section at a time (Woodson and Melcher, 1968; Zahn, 2003; Wagner et al., 2014). As EBS and tDCS were modeled based on the same static approximations, the modeling and solution procedures were equivalent, except for the source properties (e.g., location and geometry). Finally, tissue material properties (i.e., conductivity and permittivity), including those of the infarction region, were assigned impedances as detailed in Wagner et al. (2006, 2007a).

The analyses then focused on determining the current density distributions for the head models (i.e., healthy vs. infarction) and specifically determining the current density magnitude, maximum current density location in the cortex, and current density vector orientation for the EBS, TMS, and tDCS sources. Full details of the analysis are given in Wagner et al. (2004, 2006, 2007a,b, 2014).

Briefly, the stimulation source location and stimulation device orientation were normalized for the three techniques, such that the stimulation sources were located with their device source centers above the same physical target location (M1) and equally distanced along the brain surface from the modeled lesion borders, which in our case was the caudal border.

To determine the current density maximum, we ran an algorithm that scanned the current density magnitudes in the brains, and determined the magnitude and location of the maxima for the healthy head and stroke models for each stimulation source. Where the results are reported as current density magnitudes, they indicate the magnitude of the sinusoidal steady state current density for TMS and the magnitude of the steady state current densities for EBS and tDCS, all of which are provided in units of A/m^2^ unless otherwise stated.

**FIGURE 1:**
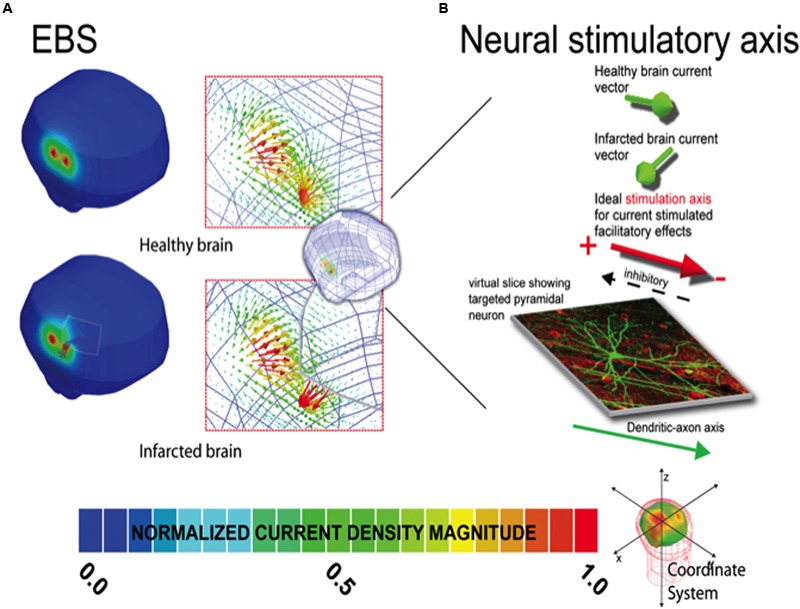
**Current density distribution maps induced by EBS stimulation.** In **(A)**, the left column depicts the current density magnitude for the corresponding healthy intact (top) and infarcted (bottom) brains stimulated with EBS. The borders and limits of the infarcted region are demarcated with a thin white line. Note that the scales in **(A)** are normalized to the maxima of the solution in each case (i.e., the maximum in the healthy brain is 1.19 A/m^2^ and 1.35 A/m^2^ in the infarcted brain). See location of the maxima in the infarcted (gray ◆) and healthy brains (gray ^∗^) indicating the location shift due to the infarction. Exact quantitative estimations on maxima shifts can be found in **Table 2**. In the right column of each panel, the vector distribution demonstrating the orientation of the currents is provided for both the intact and damaged brains. Note the direction of the currents can change substantially in the region of the perturbation. **(B)** Demonstrates how the distribution of EBS induced currents can be altered such that facilitatory stimulation might become inhibitory in select neural populations in the lesion region, when applying **subthreshold** polarizing currents where the stimulatory effect is dependent on the relative current density orientation to the axo-dendritic axis (Terzuolo and Bullock, 1956; Landau et al., 1964). In our results for select regions of tissue near the lesion border, the current orientation is altered relative to the neural axis such that the neural effect would be opposite of that predicted for the healthy brain. Note herein, the inhibitory/facilitatory axis is simplified for graphical representation, and will ultimately depend on the complexity and relative position of the neural structure, related to the axo-dendritic axis of the neuron. The total net effect across the total tissue stimulated could be comprised of a mix of areas receiving inhibitory and facilitatory stimulation (based on the relative neural cell and current density orientations in each individual patient relative to the stimulator source). Furthermore, such effects could potentially be seen in areas of in areas of complex sulcal anatomy even in healthy subjects. Unique solutions based on each individual patient’s stimulation criteria are thus recommended for individual patient dosing considerations.

The relative change between the healthy and infarcted brains is reported as the value of the difference between the current density maxima in the infarction and healthy head models divided by the current density maxima in the infarction model. Further, the individual models all shared the same Cartesian coordinate system, with an origin at the heads’ center, and thus the relative change in maxima locations between the various healthy brain and infarction models was determined by the Euclidean distance equation. The current density vector field directional patterns were also analyzed in the models, and focused on comparing the change in the current density fields’ vector orientation proximal to the current source and the lesions the healthy and infarction models [see **Figure 1**, and (Wagner et al., 2006) for further details]. The angular perturbation of the current densities between the healthy and infarction models was used to determine the relative current density orientation shift that would occur along a fixed axonal axis between the models (see **Figure 1B**). Finally, as the models were deterministic, we did not conduct statistical testing between the different solution sets.

## Results

**FIGURE 2:**
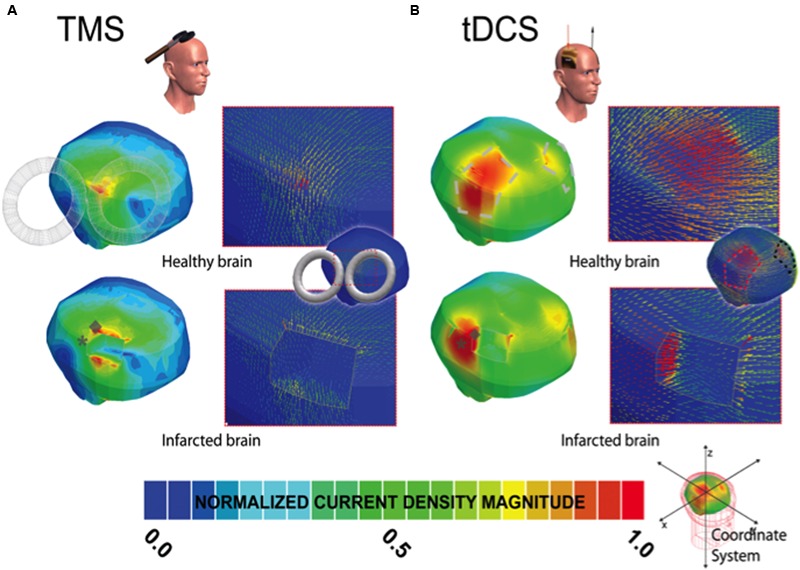
**Current density distribution maps induced by TMS and tDCS stimulation.** In **(A,B)**, the left column depicts the current density magnitude for the corresponding healthy or intact (top) and infarcted (bottom) brains stimulated with TMS and tDCS, respectively. The borders and limits of the infarcted region are demarcated with a thin white line. The modeled lesions presented for EBS (see **Figure 1A**), TMS (2A), and tDCS (2B) all have the same size and volume and occupy the exact same location in the right hemisphere in the infracted brain. As in **Figure 1A**, note that the scale of **(A,B)** is normalized to the maxima in the corresponding solution pictured (i.e., the maximum current density in the TMS healthy brain solution is 2.4 A/m^2^ and 4.16 in the infarcted brain, and 0.098 and 0.129 in the tDCS healthy and infarcted cases, respectively). The location of the maxima in the infarcted (gray ◆) and healthy brains (gray ^∗^) are both marked symbolically on the injured brain to indicate the estimated site shift (please zoom on the image for a better appreciation if needed). Note, as in EBS, the direction of the currents changes substantially in the region of the perturbation for both techniques.

Current density distributions (magnitude, location, and orientation) were altered in the presence of our idealized model of focal right frontal infarction for TMS, tDCS, and EBS, as compared to solutions in the intact brain models (**Tables 1–2** and **Figures 1–2**). For all three techniques, currents were increased in magnitude and directed toward the infarction border. Increases of peak current density in a damage brain compared to the healthy one were less drastic for EBS (+18%) than for tDCS (+32%) or TMS (+73%) (see **Table 1**). Furthermore, the vector current orientation was altered at the infarction borders, such that the net sign of the neuromodulation effects (i.e., lasting inhibition or facilitation) could be reversed (e.g., **Figure 1B** and further discussion below).

**Table 1:** Maximum current density magnitude (in A/m^2^) in the healthy and the infarcted brain.

Neurostimulation modality and polarity	Healthy brain max current density (A/m^2^)	Infarcted brain max current density (A/m^2^)	Infarcted vs. healthy brain. Relative change in max current density (%)
**EBS**			
Cathode	1.15	1.35^∗^	+17.4%^∗^
Anode	1.19	1.22	+2.50%^∗^
**tDCS**			
Anode	0.098	0.129^∗^	+31.6%^∗^
Cathode	0.082	0.084	+2.40%^∗^
**TMS**			
	2.40	4.16^∗^	+73.30%^∗^

*^∗^Corresponds to location of stimulation source proximal to the infarction border.*

**Table 2:** Coordinates of the locations (relative to the x,y,z head coordinate system) of the current density maxima in the healthy and the infarcted brain.

Neurostimulation modality and polarity	Stimulating source radius or equivalent length (mm)	Healthy brain *maxima* location x,y,z (mm)	Infarcted brain *maxima* location x,y,z (mm)	Absolute distance shift (mm)
**EBS**				
Cathode	~1 mm	53.9, 22.9, 193.8	53.1, 24.7, 197	4.0 mm^∗^
Anode	~1 mm	53.7, 6.8, 194.1	53.6, 7.2, 194.8	<1.0 mm
**tDCS**				
Anode	~25 mm	56.0, 18.2, 17.5	47.1, 27.5, 26.9	15.9 mm^∗^
Cathode	~25 mm	-14.5, 50.8, 27.3	-15.4, 50.5, 27.5	<1.0 mm
**TMS**				
	~35 mm	-4.8, -7.2, -23.1	-15.1, -20.5, -17.0	17.9 mm^∗^

*^∗^Corresponds to alterations in predicted current density maxima location if the effects of the infarction on stimulation currents were ignored.*

The overall absolute distance between the expected target and the actual site of the current maxima (comparing the healthy brain and infarction brain models) were less remarkable in overall magnitude for EBS (a 4 mm shift from the expected vs. the real maximum site) than for TMS (17.9 mm shift) or tDCS (15.9 mm shift) – see **Figures 1–2** and **Table 2**. However, relative to the size of the stimulation source, the shift of the current maxima was more drastic for EBS (~1 mm radius contacts) than for TMS (~35 mm radius contact source) or tDCS (~25 mm shortest center-edge segment for a 50 × 70 mm electrode) (see **Table 2**, and in **Figures 1A** and **2A,B**, distances between the gray ♢ and ^∗^ icons displayed on the brain models).

## Discussion

This study suggests that EBS, tDCS, and TMS neurostimulation current density distributions are altered in the presence of strokes in a manner that may explain discrepancies in CPSP treatment outcomes across the different stimulation techniques (André-Obadia et al., 2008, 2011, 2014; Hosomi et al., 2008, 2013; Lefaucheur et al., 2008, 2011a,b; Velasco et al., 2008; Tanei et al., 2011; Sachs et al., 2014). Currents flow down the path of least resistance, in the highly conductive CSF at an infarction location, and impact the current density distributions in magnitude, location, and orientation for EBS (**Figure 1**), TMS (**Figure 2A**), and tDCS (**Figure 2B**) (Wagner et al., 2006, 2007a,b, 2009).

Although the overall absolute perturbation effects in the current densities were greatest in TMS and tDCS, EBS currents were still significantly affected when the stimulatory contacts were close to irregular tissue borders of the modeled chronic stroke lesion. Moreover, the change in the location of maximal stimulation between the infarcted and healthy brains was greatest with EBS relative to the size of the stimulator (see **Figures 1** and **2**, and **Table 2**). The lower focality of TMS and tDCS, as compared to EBS, could make them less sensitive to relative mislocalizations around the targeted location. This difference could reconcile the relevance of our current findings with the fact that TMS and tDCS studies in perilesional stroke regions have generally reported beneficial therapeutic effects with potentially less variability than EBS studies (Lima and Fregni, 2008; O’Connell et al., 2014; Hosomi et al., 2015; DosSantos et al., 2016).

The altered orientation of the stimulation currents relative to the targeted neurons could impact the degree and/or the direction of inhibitory/excitatory response of the involved networks, particularly for sub-threshold stimulation conditions- see **Figure 1B** (Terzuolo and Bullock, 1956; Landau et al., 1964; Wagner et al., 2007b; Radman et al., 2009a,b; Wongsarnpigoon and Grill, 2012). The net sign of the neuromodulation effects (i.e., lasting inhibition or facilitation) could potentially be reversed in cases where the lesion boundary alters the currents’ orientation relative to the targeted cell’s axo-dendritic axis [particularly for sub-threshold stimulations (Terzuolo and Bullock, 1956; Landau et al., 1964)].

Ultimately, the varied stimulation current perturbations between the techniques could in part explain inter-technique discrepancies between tDCS, TMS, and EBS in treating CPSP. Low-intensity EBS M1 cathodic stimulation currents are postulated to affect axons parallel and superficial over the crown of the precentral gyrus (Lefaucheur, 2013). In pain treatment, maximal pain relief is postulated to be associated with late indirect waves (recorded at the spinal cord level) produced from cathodic M1 EBS and also anteroposterior M1 TMS. On the other hand, anodal M1 EBS and lateromedial M1 TMS stimulation lead to early direct waves, suggesting that the polarity and orientation of the current in these techniques activates different axonal tracts and pathways (Lefaucheur, 2016). Unlike EBS, tDCS shows more analgesic effect during anodal stimulation, potentially due to different neuronal structures being activated, or due the relative current vector orientations having similar orientations in the targeted neurons, see **Figures 1–2** (Lefaucheur et al., 2010; Lefaucheur, 2013, 2016). This suggests that the relative current-neuronal structure orientations between tDCS, TMS, and EBS should be considered when planning stimulation treatments for CPSP, especially across techniques (e.g., using TMS to predict EBS response). Proper planning of the stimulation protocol with a MRI-integrated field solver-tracking device could be helpful to address the current-tissue interactions, but only with systems that track and predict current vector orientations (i.e., systems which predict field strengths alone could not be used to overcome discrepancies between the techniques).

Although the conclusions of the current study could apply to a large number of cases, any extension of the current results to other lesion features, such as subcortical locations and single or multiple lacunar strokes, which have been explored in neurostimulation therapeutic CPSP studies, would need to be specifically evaluated for individual dosing considerations. It is clear from the present study that electromagnetic tissue properties differently affect brain stimulation dosing for different stimulation methods, and introduce a technique-dependent variability in potential therapeutic benefit. Ignoring the effects of altered neural tissue properties on the M1 stimulating currents in stroke may contribute to contradictory outcomes in CPSP neurostimulation trials (O’Connell et al., 2014; Hosomi et al., 2015). Finally, our results highlight the need for new forms of brain stimulation that can overcome these limitations and provide effective treatment for chronic pain syndromes and other disorders where brain stimulation is used.

## Author Contributions

Respective roles of each author are as follows: RR and AV-C wrote the initial version of the manuscript. AO and RA had substantial contribution in the adaptation of the final manuscript to the challenges of neurostimulation technologies and approaches in CPSP. Finally, RR, UE, LA, LD, TW, and AV-C provided substantial contribution to the design of the work, and the revised versions of the manuscript. All authors provided their final approval of the submitted version and agreed to be accountable for all aspects of the work.

## Conflict of Interest Statement

TW is the Chief Science Officer of Highland Instruments, a medical device company. He also has patents pending or issued related to imaging, brain stimulation and wound healing. All the other authors declare that the research was conducted in the absence of any commercial or financial relationships that could be construed as a potential conflict of interest.
